# The Story of the Insane from Year to Year

**Published:** 1904-10-01

**Authors:** 


### THE STORY OF THE INSANE FROM YEAR TO YEAR.

Seventeenth Annual Series.

### ROYAL ASYLUM, ABERDEEN.

On January 1st this Asylum contained 958 patients, and the number had risen to 996 on December 31st. There were 237 first admissions, 75 readmissions, 121 discharged recovered, 16 discharged relieved, 56 discharged not improved, and 81 died. The total number under treatment during the year was 1,259, and the average number resident was 973. Calculated on the admissions the recoveries were 38·7 per cent.; and calculated on the average number resident the deaths were 8·3 per cent. Of the year's deaths 30 were caused by cerebral and spinal diseases, 8 by consumption, and 10 by other lung diseases. Enteritis was the cause in one case only. Of the year's admissions 15 owed their insanity to such moral causes as domestic trouble and mental anxiety; and among the physical causes, intemperance in drink accounts for 38, hereditary influence for 57, previous attacks for 43, and in 21 no cause was ascertained. The percentage of recoveries comes up to a fair average, and yet we notice that many of the cases admitted were unfavourably placed as regards chances of recovery. For instance 25 were over 70 years of age, 2 were 7 years of age, 9 were idiotic, 12 were general paralytics, and 41 were demented. Since the Colney Hatch disaster a fire scare has run through all our asylums. Aberdeen has revised its list of precautions and appliances and has very properly added to them. Dr. Reid speaks well of the employment of nurses in the male wards, and says that all the recent admissions, the sick, and the infirm, are under their care. The Asylum has one senior assistant medical officer and two juniors.

### CAMBRIDGE COUNTY ASYLUM.

On January 1st there were 552 patients in residence, and by December 31st the number had risen to 590. There were 97 first admissions, 18 readmissions, 43 discharged recovered, 15 discharged relieved, and 51 died. The total number under treatment during the year was 587, and the average daily number resident was 564. The recoveries work out at 37·39 per cent. of the admissions, and the deaths at 8·86 per cent. of the average number resident. Of the deaths 16 were caused by cerebral and spinal diseases, 3 by consumption, and 6 by other lung diseases. In future reports we venture to hope the table of deaths will be so arranged as to group the diseases in the manner common in asylums. "meningitis" wedged between "phthisis," and "pneumonia" does not look well. Of the year's admissions 14 were supposed to owe their insanity to such moral causes as domestic trouble, adverse circumstances, and mental anxiety. Among the physical causes, intemperance in drink accounts for 17, hereditary influence for 25, and congenital defect for 27. The new wing on the female side was opened in November, and 27 patients who had been boarded out at the Three Counties' Asylum were brought home again. As the Cambridge Committee had to pay a relatively high rate of board for these cases, so they are now trying to recoup themselves by charging 16s. a week from other counties, and they will probably not have long to wait before filling the block. There is at present no better speculation than spare room in a county asylum. We are glad to notice that no post-mortem examination is made at the Cambridge Asylum unless the consent of the relatives has been obtained. There are two assistant medical officers, and one of these is a lady, an arrangement of which we cordially approve. The weekly rate of maintenance is 9s. 7½d.

### MIDDLESEX COUNTY ASYLUM AT WANDSWORTH.

One thousand four hundred and thirty-four patients were in residence at the beginning of the year, and at the end of it there was only 1 more than that number. There were 420 first admissions, 76 readmissions, 183 recoveries, 58 discharged relieved, 118 discharged not improved, and 136 died. The total number under treatment was 1,930, and the average number resident was 1,418. The recoveries were 42·1 per cent. of the admissions, and the deaths were 9 per cent. of the average number resident. Of the year's deaths 62 were caused by cerebral and spinal diseases (36 of these were from general paralysis), 16 by consumption, and 4 by other lung diseases. Of the year's admissions various moral causes account for the insanity in 94 instances, and among the physical causes the most potent were as usual drink, heredity, and previous attacks, accounting respectively for 43, 89, and 125. The asylum is somewhat overcrowded, there being 20 patients more in residence than the space is intended for. A large number of Middlesex patients is boarded out at other county asylums, but it is hoped that the new asylum will be ready next year, when the overcrowding will be ended and the cases boarded out brought back to their own county. The Committee of Visitors specially refer to the services of Dr. Rolleston, the senior assistant medical officer, and the juniors, "who by their unflagging exertions kept the asylum in a high state of efficiency during the enforced and prolonged absence of the medical superintendent." The weekly charge to the unions is 12s. 6d. per head.

